# Eco-Friendly, Simple, Fast, and Sensitive UPLC-MS/MS Method for Determination of Pexidartinib in Plasma and Its Application to Metabolic Stability

**DOI:** 10.3390/molecules27010297

**Published:** 2022-01-04

**Authors:** Essam Ezzeldin, Muzaffar Iqbal, Yousif A. Asiri, Gamal A. E. Mostafa, Ahmed Y. A. Sayed

**Affiliations:** 1 Department of Pharmaceutical Chemistry, College of Pharmacy, King Saud University, Riyadh 11451, Saudi Arabia; muziqbal@ksu.edu.sa (M.I.); gmostafa@ksu.edu.sa (G.A.E.M.); ahmedyahia009@gmail.com (A.Y.A.S.); 2 Department of Clinical Pharmacy, College of Pharmacy, King Saud University, Riyadh 11451, Saudi Arabia; yasiri@KSU.EDU.SA

**Keywords:** UPLC-MS/MS, pexidartinib, tyrosine kinase inhibitor, metabolic stability, gifitinib

## Abstract

Pexidartinib is the first drug approved by the U.S. Food and Drug Administration specifically to treat the rare joint tumor tenosynovial giant cell tumor. In the current study, a validated, selective, and sensitive UPLC-MS/MS assay was developed for the quantitative determination of pexidartinib in plasma samples using gifitinib as an internal standard (IS). Pexidartinib and IS were extracted by liquid-liquid extraction using methyl tert-butyl ether and separated on an acquity BEH C_18_ column kept at 40 °C using a mobile phase of 0.1% formic acid in acetonitrile: 0.1% formic acid in de-ionized water (70:30). The flow rate was 0.25 mL/min. Multiple reaction monitoring (MRM) was operated in electrospray (ESI)-positive mode at the ion transition of 418.06 > 165.0 for the analyte and 447.09 > 128.0 for the IS. FDA guidance for bioanalytical method validation was followed in method validation. The linearity of the established UPLC-MS/MS assay ranged from 0.5 to 1000 ng/mL with r > 0.999 with a limit of quantitation of 0.5 ng/mL. Moreover, the metabolic stability of pexidartinib in liver microsomes was estimated.

## 1. Introduction

Tenosynovial giant cell tumor (TGCT) is a rare form of joints and tendons tumors. Many patients rely on surgical treatment to remove the tumor, and more likely, it recurs over time even with surgery [1]. However, some patients are not eligible for surgical interventions, and they live with some severe symptoms, including pain, stiffness, and restricted movement. This can lead to severe disability and significantly affect the quality of their life [2].

**Figure 1:**
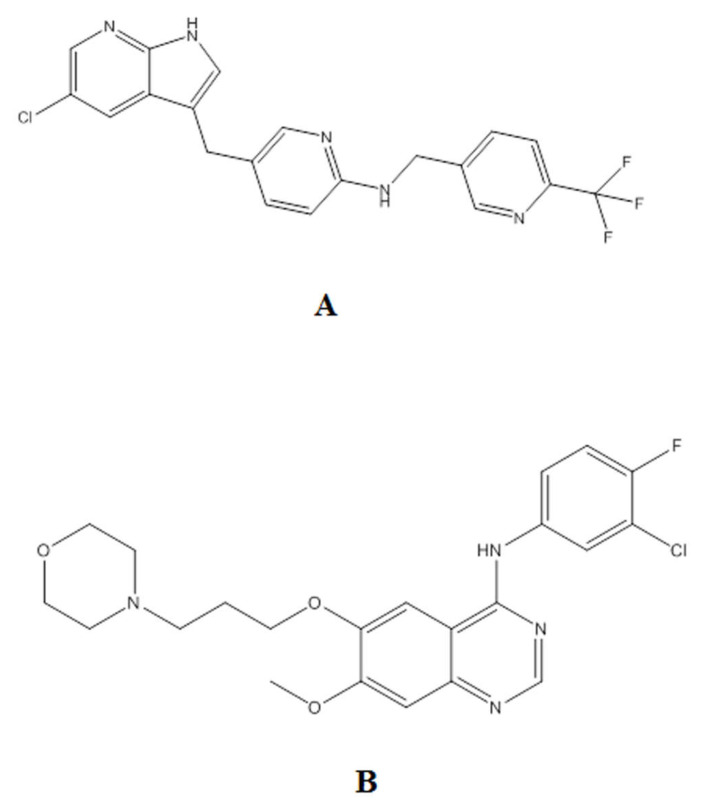
Chemical structure of PX (**A**) and IS (**B**).

Pexidartinib (PX) is a novel selective small-molecule tyrosine kinase inhibitor (Figure 1) that preferentially targets the macrophage colony-stimulating factor-1 receptor (CSF-1R). Furthermore, it inhibits closely related family members of proto-oncogene receptor tyrosine kinase (KIT) [1]. PX is the first systemic therapy approved by the U.S. FDA for the treatment of adults with symptomatic TGCT associated with severe morbidity or functional limitations and not susceptible to improvement with surgery [2]. Moreover, PX monotherapy and combination therapy tolerability were manageable in phase I/II clinical trials of patients with various malignancies, including advanced castration-resistant prostate cancer, metastatic breast cancer, advanced melanoma, refractory leukemias, and sarcoma [3].

PX recommended dose is 400 mg twice daily on an empty stomach [4]. Long-term PX treatment results in a remarkable inhibition of intra-neuronal amyloid accumulation as well as neurotic plaque deposition [5]. PX was determined to be effective and safe at a dose of 1000 mg/day; however, some adverse events were observed, including anemia and high levels of bilirubin and liver enzymes [6]. Because of the risk of serious liver injuries, PX is restricted for use through a drug safety program known as Risk Evaluation and Mitigation Strategy that is designed to assess medications’ benefit/risk ratio [7]. Hence patients’ liver function should be closely monitored prior, and during PX treatment [8,9,10].

PX pharmacokinetics showed moderate absorption with maximum concentration achieved at 2–4 h. PX is highly bound to plasma protein (>99%) and highly metabolized, mainly by CYP3A4 (cytochrome 3A4) and UGT1A4 gene. PX is considered a moderate inducer of GYP3A and a weak inhibitor for CYP2C9 (cytochrome C29). A recent drug-drug interaction study demonstrated that PX co-administration with midazolam resulted in a decrease in area under the time-concentration curve (AUC) of midazolam by 21%. However, PX co-administration with digoxin increased digoxin maximum concentration by 32%. Moreover, omeprazole exposure was reduced when co-administered with a single oral dose of PX [11]. On the other hand, it was found that PX metabolism is inhibited by co-administration of fluconazole and itraconazole that leads to an increase in PX concentration. However, other antifungal such as isavuconazole and posaconazole has no effects on PX metabolism. Therefore, therapeutic drug monitoring of PX during its concurrent use with fluconazole or itraconazole is essential [12]. PX can be safely administered with sirolimus [13]. It has been reported that PX concurrent treatment with docetaxel potentiates the suppression of tumor growth induced by docetaxel [14]. Additionally, age, sex, and race did not have a significant impact on PX pharmacokinetics [1]. A phase I dose-escalation study in pediatric patients with relapsed and refractory leukemia and solid tumors showed that PX was well tolerated at three different dose levels (400 mg/m^2^, 600 mg/m^2,^ and 800 mg/m^2^) for 28-day cycles with no observed dose-limiting toxicities. Moreover, PX pharmacokinetics was linear over the three dose levels [15].

Few studies have been recently published for the quantitative determination of PX in pharmaceutical products using HPLC [16], in plasma to study PX population pharmacokinetics [17] and pediatric pharmacokinetics/ pharmacodynamics [15], in cerebrospinal fluids to measure its brain uptake [18] and in drug-drug interaction, studies using LC-MS/MS [11]. However, these studies lack the details of the methods chromatographic parameters and extraction techniques. Moreover, the sensitivity of these methods is low. In this study, we developed and validated a sensitive, accurate, and rapid UPLC-MS/MS method for the quantification of PX in human plasma using a simple liquid-liquid extraction (LLE) procedure. The use of UPLC not only increases separation throughput but also reduces the retention time and volume of solvents required for the separation, which is useful for the greener approach of the separation method.

## 2. Results and Discussion

### 2.1. Method Validation

#### 2.1.1. Optimization of Mass Spectrometry Conditions

**Figure 2:**
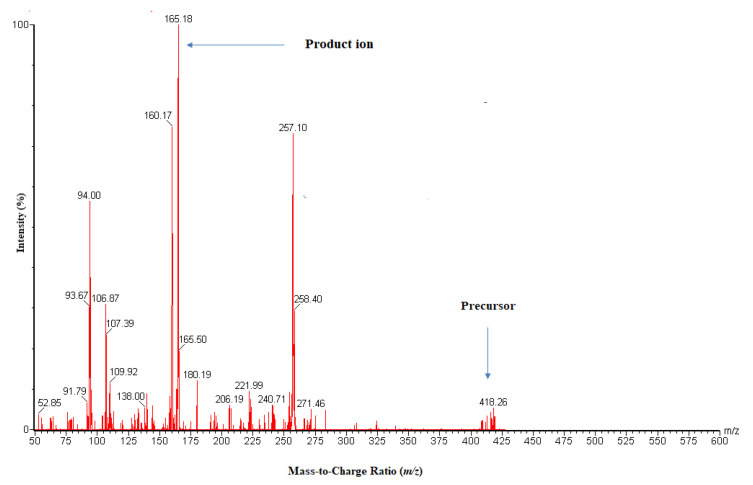
Typical representation of precursor to product ion spectra of PX- in ESI-positive ionization mode.

During MS/MS optimization, each of PX and IS (400 ng/mL) was directly infused into a mass spectrometer using turbo ion spray as the ionization source. The highest sensitivity for PX and IS was achieved in ESI-positive mode, and the protonated molecular ions were *m*/*z* 418.06 and 447.09, respectively. Following fragmentation, the product ions with the highest intensity were produced at *m*/*z* 165.0 and 128.0 for PX and IS, respectively. Therefore, the MRM ions transition of *m*/*z* 418.06 → 165.0 for PX and *m*/*z* 447.09 → 128.0 for IS were selected for their identification and quantification Figure 2. The optimum conditions for MS/MS parameters we adjusted and summarized in Table 1.

#### 2.1.2. Optimization of Extraction and Chromatographic Methods

The mobile phase is one of the most significant parameters in chromatographic analysis. The components of the mobile phase can have a high impact on analytes separation, retention time, and sensitivity of the method. This may suppress or promote the ionization of the molecules that are analyzed [19,20]. Different ratios of organic solvents (methanol/acetonitrile), formic acid at different strengths, and various buffer concentrations, including ammonium citrate and ammonium format, were investigated. In addition, different columns were tested to select the column with optimum sensitivity, resolution, and retention time. Finally, the best chromatographic separation was achieved using an acquity BEH C_18_ column (2.1 mm × 100 mm, 1.7 μm, Waters Corp., Milford, MA, USA) at 40 °C. The mobile phase consisted of 0.1% formic acid in acetonitrile: 0.1% formic acid in water (70:30), and the flow rate was 0.25 mL/min. LC-MS/MS is the most common bioanalytical method used now because it is robust and sensitive. Detection of the mass spectrometry method relies on mass-to-charge ratio, and the baseline separation of peaks is generally being not required in LC-MS on the contrary of conventional LC with UV detection due to the non-specific nature of this detection [21]. This distinctive advantage of the separations by LC-MS/MS allows for simultaneous quantitation of active pharmaceutical ingredients in plasma [22]. In the proposed method, the analyte and IS have similar physiochemical properties and elute at approximately 1.0 min. as mass spectrometer separated them based on their mass-to-charge ratio (*m*/*z*). This allowed separation and quantitation of the analyte and IS in a short run time.

Several techniques were tested to extract PX and IS from human plasma. The protein perception method was investigated using methanol and acetonitrile. Although the method was easy and fast, the recovery was low. The liquid-liquid extraction technique was also tested using different organic solvents (e.g., diethyl ether, dichloromethane, tert-butyl methyl ether, and ethyl acetate). The optimum extraction method was achieved using 50 µL of acetonitrile followed by 1 mL of *tert-butyl methyl ether*. The extraction tubes were then centrifuged at 10,500× *g* for 5 min at 8 °C.

Several techniques were tested to extract PX and IS from human plasma. The protein perception method was investigated using methanol and acetonitrile. Although the method was easy and fast, the recovery was low (34%). The liquid-liquid extraction technique was also tested using different organic solvents, including diethyl ether, dichloromethane, tert-butyl methyl ether, and ethyl acetate. The recovery obtained using these solvents was 65.3%, 58.4%, 72.4%, and 68.1%, respectively. The optimum extraction method was achieved using 50 µL of acetonitrile followed by 1 mL of tert-butyl methyl ether. The extraction tubes were then centrifuged at 10,500× *g* for 5 min at 8 °C.

#### 2.1.3. Selectivity and Specificity

**Figure 3:**
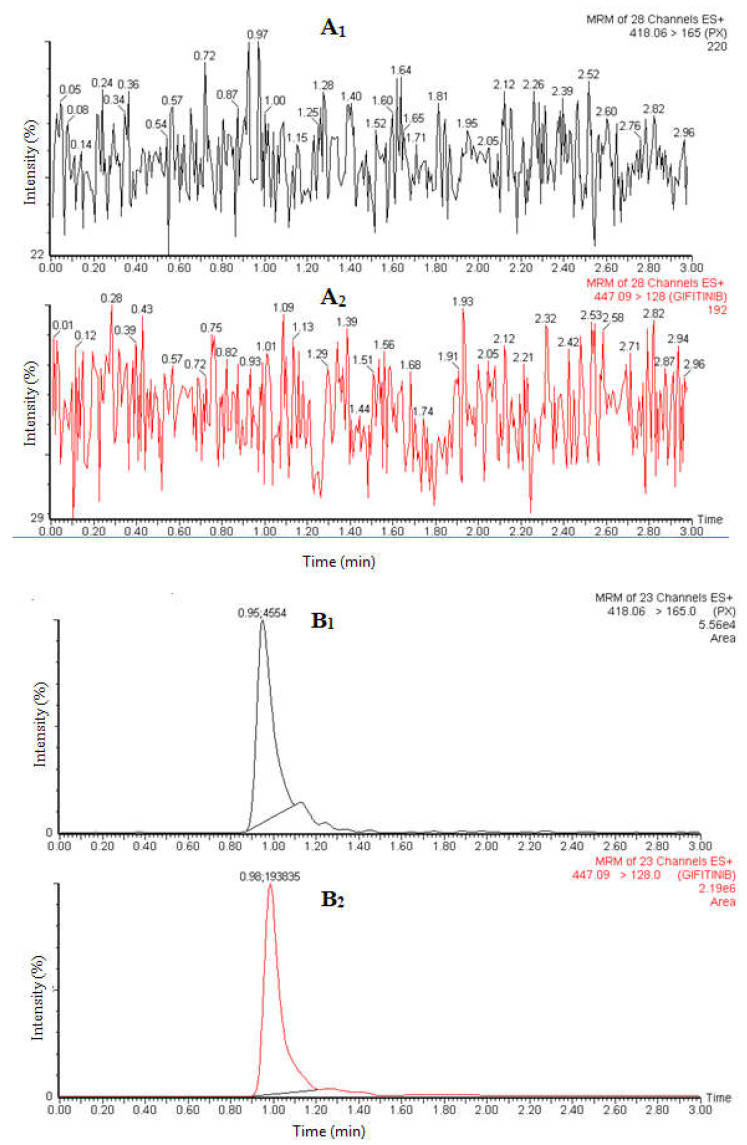
MRM chromatograms of PX and internal standard in blank plasma (**A**), and plasma spiked at LLOQ (**B**).

Selectivity and specificity are the ability of an analytical method to differentiate and quantify the analyte in the presence of other components in plasma samples. Figure 3 shows the blank chromatograms of the analyte and IS, and chromatograms of plasma samples spiked at LLOQ (low limit of quantitation). The absence of endogenous peak and/or MS response at the retention times of the analyte and the IS was considered as evidence for the selectivity of the method.

#### 2.1.4. LLOQ and the Linearity of Plasma Calibration Curve

Three calibration curves were constructed in human plasma using nine different concentrations in the range of 0.5–1000.0 ng/mL. Suitable linearity was obtained with a coefficient of variation (r) of ≥0.999. A weighted (1/x^2^) linear regression of PX was used for the back-calculation of concentrations for all points. The sensitivity of this method was established by the LLOQ at 0.5 ng/mL that exhibited a high signal-to-noise (S/N) ratio with precision ≤ 20% and accuracy (±20%) (Table 2, Figure 3). The low limit of detection (LOD) was 0.2 ng/mL.

#### 2.1.5. Accuracy and Precision

The intra- and inter-day accuracy and precision were evaluated using six replicates of QC samples at LLOQ, LQC (low-quality control concentration), MQC (medium quality control concentration), and HQC (high-quality control concentration). The values of intra-day and inter-day precision were found to be ≤13.12% and ≤5.58%, respectively, whereas the intra-day and inter-day accuracy were within 82.80–90.66% and 82.57–92.63%, respectively (Table 2).

#### 2.1.6. Recovery and Matrix Effects

The extraction recovery of PX from the plasma matrix was assessed by comparing the analytical results of extracted QC samples with corresponding extracts of blanks spiked with the analyte post extraction.

The matrix effect was evaluated by comparing peak area ratios of PX spiked into the extracted blank matrix to the neat reference standard solutions. Results of the assessment of extraction recovery and matrix effect are presented in Table 3.

The mean recovery value of three QC concentrations (1.5, 150, and 750 ng/mL) was 84.23%, with an RSD% of 2.47. The matrix effect of PX was ranged from 87.54% to 89.46%, indicating no significant effect was observed of the endogenous plasma materials on the entire procedure. Extraction recovery and matrix effect for IS were 76.50% and 89.20%, respectively (Table 3).

#### 2.1.7. Stability

The stability of the analyte was assessed in plasma samples in anticipated storage conditions using five replicates of two QC levels, low (15.0 ng/mL) and high (750.0 ng/mL). Short- and long-term stability was investigated after storage at room temperature (about 24 °C) for 8 h and at −80 °C for 60 days, respectively. Moreover, auto-sampler stability was evaluated for 24 h at 12 °C. Three complete freeze-thaw cycles (from −80 °C to room temperature) were also assessed. All results passed the requirement of accuracy (±15%) and precision (≤15%) for both low and high QC concentrations (Table 4).

### 2.2. In Vitro Metabolic Stability Study

**Figure 4:**
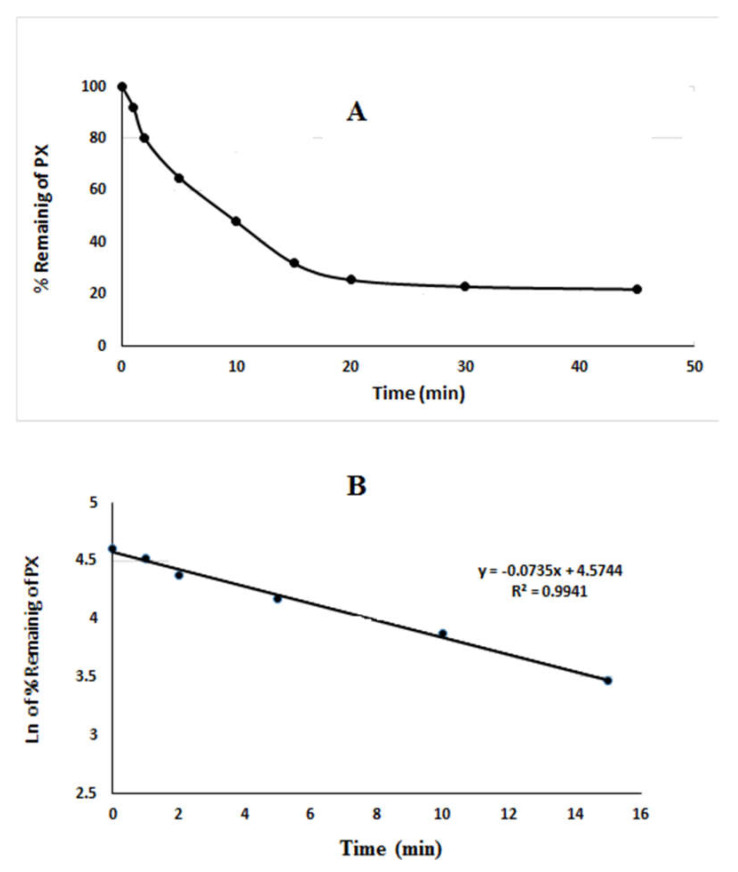
The metabolic stability curve of PX in liver microsomes (**A**) and the regression equation of the linear part of the curve (**B**).

After sample analysis, PX metabolic stability curve was established using the generated data. The PX percent remaining is plotted (Y-axis) versus incubation time (X-axis) (Figure 4A). From this plot, the time points in the linear range were chosen to plot the natural logarithm of the percent parent compound remaining versus time (Figure 4B). Equation of the linear part of the curve was y = −0.0735X + 4.5744 with R² = 0.9941. The slope of the linear part gives the rate constant for the disappearance of PX that is required to calculate vitro t_1/2_ and intrinsic clearance [23,24]. The obtained data showed that the t_1/2_ and Cl_int_ were 9.84 min and 37 µL/min/mg, respectively.

### 2.3. Greenness of the Method

**Figure 5:**
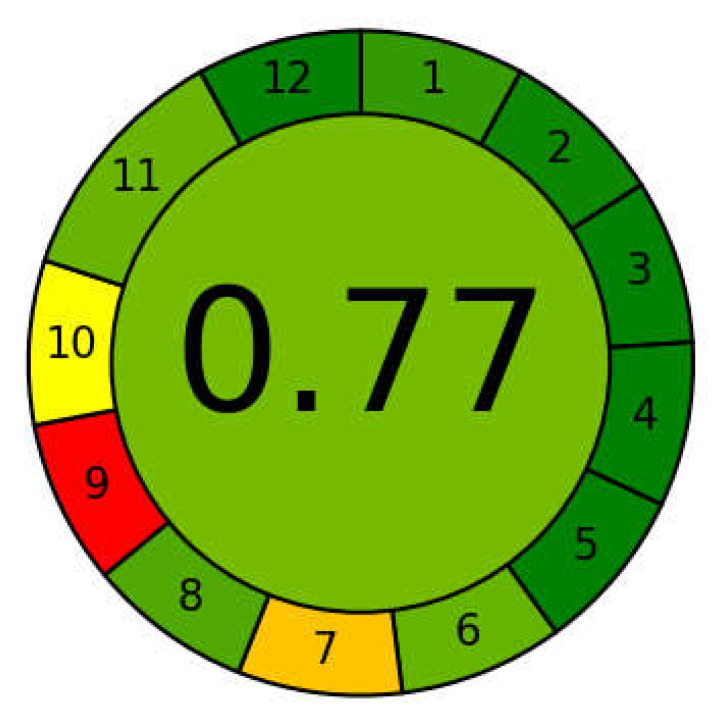
Agree pictogram for evaluation of the proposed method greenness.

The greenness of the proposed methods was evaluated applying for Eco-Scale proposed by Van-Aken et al. [25] and by using AGREE software. The results obtained by applying the Eco-Scale of the methods indicated in Table 5, and the score was found to be 80, represents an excellent green analysis. Moreover, the greenness of the method checked by AGREE software was compatible with these results. The final score, as indicated AGREE pictogram (Figure 5), was found to be 0.77. Therefore, the proposed method can be considered as an excellent green method for the quantification of PX in plasma. Moreover, In the proposed method, the amount of waste was less than 50 g, and none of the used solvents are listed in the PBT (persistent, bioaccumulative, and toxic) list. In addition, they are not corrosive or hazard indicating that this method agrees with the greenness profile criteria according to the emergency planning and community right-to-know act, 2004 [26].

## 3. Materials and Methods

### 3.1. Chemicals and Reagents

PX (purity 97%) was purchased from MEC Med Chem. Express LLC, (Middlesex, NJ, USA). IS (purity %) was purchased from Beijing Mesochem (Beijing, China), and dimethyl sulphoxide (DIMSO) was obtained from Sigma-Aldrich (Darmstadt, Germany). Acetonitrile (HPLC grade), formic acid, and methyl tert-butyl ether were purchased from Sigma-Aldrich (St. Louis, MO, USA). Purified water was obtained from the Milli Q purification system, Millipore (Bedford, MA, USA). Drug-free human plasma was kindly provided from King Khalid University Hospital (Riyadh, Saudi Arabia). Human microsomes (HLMs) of 50 mixed-sex donors were purchased from Thermo Scientific (Waltham, MS, USA).

### 3.2. Stock Solution, Calibration Standards, and Quality Control Sample Preparation

PX and IS (1.0 mg/mL) standard stock solutions were prepared separately in DIMSO. PX and GI standard solutions were then diluted in methanol to provide final concentrations of 100.0 µg/mL. Intermediate serial standard solutions were prepared in 50% methanol to be used for preparation of 9 calibration standards in plasma (0.5, 1.0, 5.0, 10.0, 50.0, 100.0, 200.0, 500.0, 1000.0 ng/mL). Similarly, four plasma QC samples (0.5, 1.5, 150.0, and 750.0 ng/mL) were designed as LLOQ, low QC, middle QC, and high QC, respectively. All spiked samples were stored at −80 °C. It is noteworthy to mention that gefitinib was chosen as an internal standard because it is chemically similar to the analyte and compatible with the analyte chromatographic separation and extraction method [27].

### 3.3. Sample Preparation

Calibrators and QC samples were prepared using the liquid-liquid extraction technique. In 2 mL Eppendorf tubes, 10 µL of IS (100.0 µg/mL) was added to 100 µL of calibrators and QC plasma samples. Then 50 µL of acetonitrile was added to all tubes. After vortex-mixing for 1 min, 1 mL methyl tert-butyl ether was added. All tubes were mixed for 20 min using a laboratory orbital shaker and then centrifuged at 10,400× *g* at 8 °C for 10 min. The supernatant was then transferred to 5 mL test tubes and evaporated using a vacuum concentrator at 40 °C. The residues were reconstituted in 100 μL of mobile phase, and a 5 μL was injected into the LC-MS/MS system.

### 3.4. Chromatographic Conditions

PX and IS were eluted on an acquity BEH C_18_ column (2.1 mm × 100 mm, 1.7 μm, Waters Corp., Milford, MA, USA) kept at 40 °C. The mobile phase used for the analysis was composed of 0.1% formic acid in acetonitrile: 0.1% formic acid in water (70:30), and the flow rate was 0.25 mL/min. The auto-sampler temperature was maintained at 12 °C.

### 3.5. Mass Spectrometry

A UPLC-MS/MS system consisting of a water acquity triple quadrupole tandem mass spectrometer and ultra-performance liquid chromatography (Milford, MA, USA) was used in this study. The detection was performed using multiple reaction monitoring (MRM) in electrospray-positive ion mode. The MRM transitions selected and mass optimization parameters are summarized in Table 1. The system was operated by Masslynx 4.1 software, and data were processed using the Target Lynx™ program.

### 3.6. Method Validation

The developed assay was validated in compliance with the U.S. FDA guidelines for the validation of bioanalytical methods [28]. Selectivity, low limit of detection (LOD), LLOQ, linearity, accuracy, precision, recovery, matrix effect, and stability at different storage conditions were established during the method validation process.

### 3.7. In Vitro Metabolic Stability

In vitro metabolic stability method is an early estimation and prediction of in vivo metabolism of the drug [29]. PX undergoes metabolism through different phases, including mainly oxidation with CYP3A4 and glucuronidation by UGT1A4. In this study, the metabolic stability method described by Hill (2004) [30] was applied. Hepatic microsomes was used for the measurement of PX stability as it is the source of major enzymes responsible for drug metabolism besides other enzymes that contribute to drug metabolism [31].

To a test tube containing 5 µL of PX (5 µg/mL), 450 µL of warm phosphate buffer was added and warmed at 27 °C. Following the addition of 20 µL of freshly prepared NADPH (20 mM), the sample was shaken in a water bath for 5 min at 37 °C. To initiate the reaction, 5 µL of microsomes (0.5 mg/mL) was added. The reaction was continued in a shaking water bath, and the temperature was kept at 37 °C. The reaction was terminated by adding 250 µL of acetonitrile containing IS at a time interval of 0.0, 2.0, 5.0, 10.0, 15.0, 30.0, and 45.0 min. Following sample centrifugation at 10,500× *g* for 5 min at 8 °C, the clear supernatant was transferred to auto-sampler vials, and 5 µL were injected onto UPLC-MS/MS. The calibration curve of PX was prepared in phosphate buffer at the same concentrations curve of spiked plasma. PX stability was assessed by measuring its turnover. From the analysis results, the percentage of the remaining PX is plotted versus time.

To achieve the linearity between the ratios of metabolism versus the incubation time, PX was used in a concentration of 5 µg/mL (below the Michaelis–Menten constant). The metabolic stability curve was constructed between incubation times (x-axis) against the percentage remaining PX. From the constructed curve, the concentrations that exhibited linearity (0–15 min) were selected to plot another curve of time versus natural logarithm (Ln) PX remaining. The slop of the linear portion represented the rate constant [32]. From the plotted curve, the in vitro t1/2 was calculated using the following equation

$$\mathrm{In}\;\mathrm{vitro}\;t1/2=\frac{\ln2}{\mathrm{Slope}}$$

The intrinsic clearance was calculated using the following equation [32]:

$$\mathrm{CL}\;\mathrm{int}=\frac{0.693}{\mathrm{in}\;\mathrm{vitro}\;t1/2}.\;\frac{\mu L\;\mathrm{incubation}}{\mathrm{mg}\;\mathrm{microsomes}}$$

### 3.8. Greenness of the Method

The concept for Eco-Scale of Van-Aken et al. [25] was applied to evaluate the greenness of the current analytical methods, which provides information on the whole procedure, including parameters, from sampling through transport, storage, and sample preparation to final determination. This concept uses a scale from 0 to 100, with 0 representing a totally failed reaction (0% yield) and 100 representing the ideal reaction. Additionally, the greenness of the method was also performed using AGREE software [33]. The method greenness assessment by AGREE is taken from the twelve significance principles of green analytical chemistry (GAC) [34] and is transformed into a unified 0–1 scale. The result is a pictogram indicating the final score.

## 4. Conclusions

An analytical UPLC-MS/MS assay was described and validated for PX determination in plasma. The developed method exhibited suitable sensitivity and selectivity, high throughput sample analysis, and a wide calibration range (0.5–1000.0 ng/mL) that is appropriate for preclinical pharmacokinetic and toxicokinetic studies. Furthermore, this method can be described as a green chemistry approach with the use of a low volume of organic solvent (acetonitrile), and it is more sensitive than the previously published methods. The proposed method was successful in the estimation of PX metabolic stability in human liver microsomes.

## Figures and Tables

**Table 1:** Mass optimization parameters for PX and gifitinib (IS).

Parameters	PX	Gifitinib (IS)
**I. Compound Parameters**		
Precursor	418.06	447.09
Product ion	165.0	128.0
Dwell time (s)	0.005	0.05
Cone voltage (V)	50	40
Collision energy eV	34	24
**II. Instrument Parameters**		
Collision gas flow rate (mL/min)	0.1	0.1
Nitrogen flow rate	600 L/h	600 L/h
Source temperature (°C)	150	150
Desolvation temperature (°C)	350	350

**Table 2:** Inter- and intraday precision and accuracy of PX in plasma.

Conc. (ng/mL)	Interday			Intraday		
	Mean ± SD	Precision (CV %)	Accuracy (%)	Mean ± SD	Precision (CV %)	Accuracy (%)
0.5	0.41 ± 0.04	9.75	82.57	0.42 ± 0.033	7.85	82.80
1.5	1.39 ± 0.12	8.63	92.63	1.36± 0.09	7.14	90.66
150	127.85 ± 11.48	9.26	85.23	130.02 ± 3.36	2.58	86.68
750	664.91 ± 37.10	5.58	88.65	644.16 ± 84.57	13.12	85.88

**Table 3:** Recovery % and matrix effects of PX and IS in plasma.

Compound	Nominal Conc. (ng/mL)	Extraction Recovery			Matrix Effects
		Mean	RSD *	Mean	RSD *
		(%)	(%)	(%)	(%)
PX	1.5	85.90	5.4	88.50	12.68
	150	85.40	2.10	87.54	5.55
	750	81.4	13.3	89.46	10.01
IS	100	76.5	6.5	89.20	8.90

* RSD: relative standard deviation.

**Table 4:** Stability of PX in plasma under different storage conditions.

Stability Type	Nominal Con. (ng/mL)	Measured Con. (ng/mL)	Precision (%)	Accuracy (%)
Short-term	150	131.5 ± 9.2	7.07	87.66
	750	652.21 ± 32.44	4.97	86.96
Long-term	150	129.9 ± 9.9	7.62	86.6
	750	665.39 ± 63.04	9.47	88.71
Thaw and freeze	150	129.8 ± 10.3	7.94	86.53
	750	647.52 ± 73.69	11.38	86.33
Auto-sampler (24) h	150	132.5 ± 12.9	9.74	86.33
	750	638.60 ± 41.00	6.42	85.15

**Table 5:** The penalty points of the method for the determination and quantitation of the PX in plasma.

Parameter	Value	Penalty Points
Dimethyl sulfoxide	<10 mL (g)	1
Acetonitrile	<10 m L (g)	4
Methanol	<10 mL (g)	6
Ammonium acetate	<10 mL (g)	1
Mmethyl tert-butyl ether	<10 mL (g)	3
Waste	1.0 mL /run (g)	3
Instrument energy	More than 1.5 kw/h	2
Total penalty points		20
Eco-scale score		80
